# Bioinformatics Analysis of ZBTB16 as a Prognostic Marker for Ewing's Sarcoma

**DOI:** 10.1155/2021/1989917

**Published:** 2021-10-06

**Authors:** Ke Ding, Wenli Qiu, Dianbo Yu, Huade Ma, Kangqi Xie, Fuqiang Luo, Shanlang Li, Zaiyong Li, Jihua Wei

**Affiliations:** ^1^Department of Sports Medicine, Affiliated Hospital of Youjiang Medical University for Nationalities, Baise, Guangxi, China; ^2^Department of Lab Medicine, Affiliated Hospital of Youjiang Medical University for Nationalities, Baise, Guangxi, China

## Abstract

**Objective:**

The purpose of this study is to identify novel biomarkers for the prognosis of Ewing's sarcoma based on bioinformatics analysis.

**Methods:**

The GSE63157 and GSE17679 datasets contain patient and healthy control microarray data that were downloaded from the Gene Expression Omnibus (GEO) database and analyzed through R language software to obtain differentially expressed genes (DEGs). Firstly, Gene Ontology (GO) and Kyoto Encyclopedia of Genes and Genomes (KEGG) functional enrichment, protein-protein interaction (PPI) networks, and Cytoscape Molecular Complex Detection (MCODE) plug-in were then used to compute the highest scores of the module. After survival analysis, the hub genes were lastly obtained from the two module genes.

**Results:**

A total of 1181 DEGs were identified from the two GSEs. Through MCODE and survival analysis, we obtain 53 DEGs from the module and 29 overall survival- (OS-) related genes. ZBTB16 was the only downregulated gene after Venn diagrams. Survival analysis indicates that there was a significant correlation between the high expression of ZBTB16 and the OS of Ewing's sarcoma (ES), and the low expression group had an unfavorable OS when compared to the high expression group.

**Conclusions:**

High expression of ZBTB16 may serve as a predictor biomarker of poor prognosis in ES patients.

## 1. Introduction

Ewing's sarcoma (ES) is the second most common sarcoma of bone and soft tissue, usually occurring in children and adolescents [1]. The typical genetic feature is EWSR1 gene translocation, which leads to EWS-FLI1 gene fusion in t(11;22) [2], accounting for 85% of the ES family [3, 4], and is dominated by small round cells [5]. The early data report of the National Cancer Institute showed that the incidence of ES differed by gender, age, and race. Males accounted for 60.3%, mostly under 24 years old, and the incidence of whites was nine times that of African-Americans [6].

At present, optimal treatment of ES emphasizes multimodal therapy [7–9]. Chemotherapy is an efficient treatment for ES, and it significantly improves the 5-year overall survival (OS) rate [10, 11]. However, due to the lack of early efficient diagnosis, the high recurrence rate and distant metastasis of patients with ES lead to a poor prognosis [12], and the prognosis of patients with ES is not optimistic. Patients with ES showed poor survival in age ≥ 18 years, tumor size > 10 cm, receiving radiotherapy alone, and receiving no treatment [13]. Therefore, there is still an urgent need for new diagnoses and treatment strategies to improve long-term survival.

With the development of tumor genomics research such as high-throughput sequencing, it is of great significance to find potential prognostic treatment pathways and targets for ES. In this study, our purpose is to investigate the biomarkers related to the prognosis of ES. We processed and analyzed microarray data from GSE63157 and GSE17679 datasets from the Gene Expression Omnibus (GEO) database. Gene Ontology (GO) and Kyoto Encyclopedia of Genes and Genomes (KEGG) functional and pathway enrichment analysis, protein-protein interaction (PPI) network construction of pivot genes, and identification of key genes that are significantly related to OS in ES are aimed at providing therapeutic strategies for the prognosis of ES.

## 2. Materials and Method

### 2.1. Data Source and Data Processing

Based on the GEO database (https://www.ncbi.nlm.nih.gov/geo/), we obtain two microarray datasets of ES: GSE63157 and GSE17679. GSE63157 was submitted by Volchenboum SL and Andrade J et al. and GSE17679 by Savola S and Klami A et al. GSE63157 included 85 ES patient samples; GSE17679 consisted of 88 ES patient samples and 18 matched human normal skeletal muscle samples (10 ES cell line samples were deleted). The R GEOquery package (version 2.60.0) does the download annotation. GSE63157 was sampled based on the GPL5715 Affymetrix Human Exon 1.0 ST Array. GSE17679 was sampled based on the GPL570 Affymetrix Human Genome U133 Plus 2.0 Array.

### 2.2. Identification of DEGs

All the samples' data of GSE63157 and GSE17679 were normalized and visualized by the R “Limma” package (version 3.48.1) and also the annotation. The screening conditions for the DEGs were an absolute value of log2FC > 2 and adj. *P* value < 0.05.

### 2.3. GO and KEGG Enrichment Analysis

The R “Clusterprofile” package (version 4.0.0) was used for GO and KEGG enrichment analysis and visualization. GO analysis was used to annotate the biological process (BP), cytological component (CC), and molecular function (MF) of DEGs, and KEGG enrichment analysis was used to understand the related signaling pathways; *P* < 0.05 was statistically significant.

### 2.4. PPI Network Construction and Module Analysis

The online tool STRING (http://string-db.org) (version 11.0) was used to construct the PPI network of DEGs. The threshold for statistically significant interaction is a confidence score > 0.9. Cytoscape (version 3.6.1) is software providing an open platform for the visualization of biological processes, molecular interaction networks, and integration of these networks. Molecular Complex Detection (MCODE) (version 1.5.1), a plug-in of Cytoscape, is applied to discover densely connected regions and identify the significant modules through clustering a given network. MCODE scores > 10, *k*‐score = 2, max depth = 100, node score cut‐off = 0.2, and degree cut‐off = 2 were set as the criteria for the selection of significant modules.

### 2.5. Survival Analysis of Hub Gene

The survival data of 173 ES patients samples were integrated with the expression data of DEGs. Univariate and multivariate Cox regression analyses were performed through using the R “Survival” package to identify the DEGs associated with the OS time and clinical characteristics of ES, and the hub genes that could independently guide the prognosis were found by taking the overlap with module genes. The ES patients' samples were divided into the high expression groups and the low expression groups. The difference between the hub genes and ES was further verified by the Kaplan-Meier survival curve. A Venn diagram was used for visualization, and *P* < 0.01 was statistically significant.

## 3. Results

### 3.1. Normalization of Total DEGs

**Figure 1:**
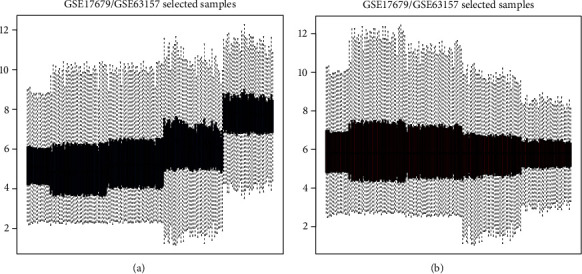
Normalization of ES patients' samples: (a) before normalization of total DEGs; (b) following normalization of total DEGs.

Gene expression data of GSE63157 and GSE17679 were standardized and displayed in a boxplot. The black line in (Figure 1(b)) is basically at the same level, indicating a high consistency of data.

### 3.2. Identification of DEGs

**Figure 2:**
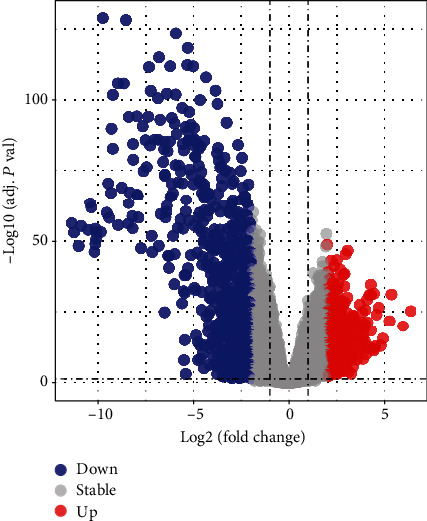
Screening of DEGs: (a) the volcano plot showed DEGs between ES groups and normal control groups. Grey represents the not significant change in expression. Red represents upregulation. Blue represents downregulation.

A total of 1181 DEGs were identified from GSE63157 and GSE17679 datasets, of which 364 were upregulated genes and 817 were downregulated genes (Figure 2(a)). Volcano plot of the DEGs, respectively, suggests that the expression of identified DEGs can correctly distinguish the case and normal samples.

### 3.3. GO and KEGG Enrichment Analysis

**Figure 3:**
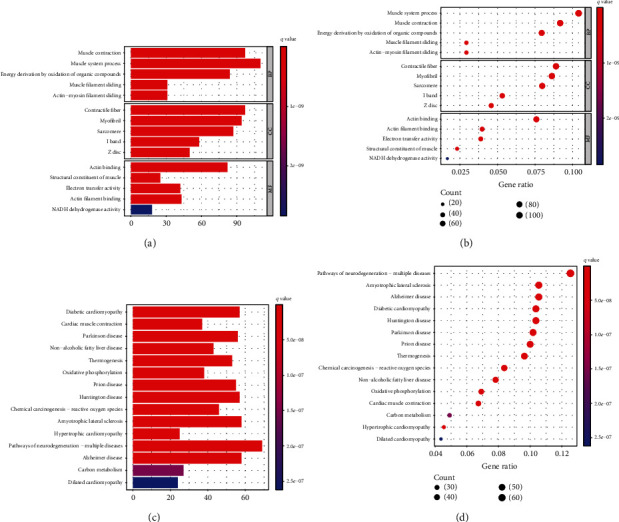
GO and KEGG pathway analysis of the DEGs. The color depth of nodes refers to the *q*-value. The size of nodes refers to the number of genes. (a) Barplot of top 5 significantly enriched biological processes, cell component, and molecular function in DEGs. (b) Bubble plot of top 5 significantly enriched biological processes, cell component, and molecular function in DEGs. (c) Barplot of top 15 significantly enriched molecular functions in DEGs. (d) Bubble plot of top 15 significantly enriched KEGG pathways in DEGs.

The R package “Clusterprofile” was used for functional enrichment analysis to assess the biological classification of DEGs (Figure 3). GO analysis showed (Figures 3(a) and 3(b)) that among biological processes, DEGs were significantly involved in the muscle system process (110 genes), muscle contraction (97 genes), energy derivation by oxidation of organic compounds (84 genes), actin-mediated cell contraction (49 genes), and muscle filament sliding (31 genes). In the cell component, DEGs were significantly involved in contractile fiber (97 genes), myofibril (94 genes), sarcomere (87 genes), I band (58 genes), and Z disc (50 genes). In molecular function, DEGs are significantly involved in binding-related projects. The molecular function includes actin binding (82 genes), structural constituent of muscle (25 genes), electron transfer activity (42 genes), actin filament binding (43 genes), and NADH dehydrogenase activity (18 genes). In addition, KEGG analysis showed (Figures 3(c) and 3(d)) that DEGs were mainly involved in diabetic cardiomyopathy (57 genes), cardiac muscle contraction (37 genes), and pathways of neurodegeneration-multiple diseases (69 genes) (Table 1).

### 3.4. PPI Network Construction and Module Analysis

**Figure 4:**
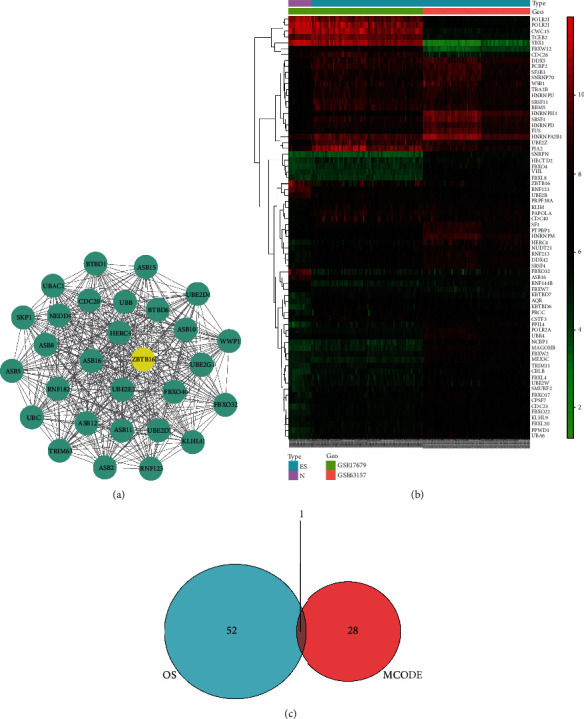
Screening of hub gene. (a) The most significant module was obtained from the PPI network with 29 nodes and 406 edges. The hub gene ZBTB16 was signature in yellow. (b) Heatmap of the top 72 differentially expressed genes in patients with ES compared with those in a normal cohort. Red represents upregulation. Green represents downregulation. (c) Venn diagram of the hub genes. The overlapping one gene is the hub gene.

With a total of 645 nodes and 3300 edges, the PPI network information of the DEGs from STRING was imported into Cytoscape, and the module of DEGs was constructed. We just take the module with the highest MCODE score. This module contains 29 nodes and 406 edges (Figure 4(a)).

### 3.5. Hub Gene Screening and Identification

Survival analysis showed that there was a total of 72 DEGs related to OS time of patients with ES (Figure 4(b)), among which 53 genes were related to clinical characteristics. The hub gene was obtained after the intersection with 29 genes in the module with the highest MCODE score (Figure 4(c)).

### 3.6. Survival Analysis of Hub Gene

**Figure 5:**
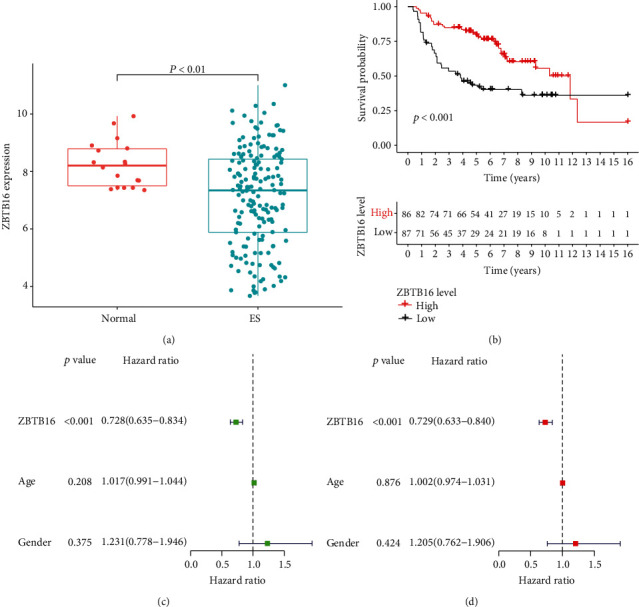
Survival curves of the hub gene (Kaplan-Meier plotter). (a) ZBTB16 expression in normal and ES patient tissues. Scatter plot indicating a higher expression of normal tissues when compared to ES patients (*P* < 0.01). (b) Kaplan-Meier survival curves for ZBTB16 in the high and low expression groups (*P* < 0.001). (c) Univariate Cox regression analysis for ZBTB16 (*P* < 0.001). (d) Multivariate Cox regression analysis for ZBTB16 (*P* < 0.001).

A total of 173 ES patients have follow-up information for survival analysis. All the ES patients were divided into the high expression group (*n* = 87) and the low expression group (*n* = 86), according to the median expression levels of ZBTB16. The expression of ZBTB16 was downregulated in the ES patients group, and the OS time of patients with low expression was poorer than the high expression group. Univariate and multivariate Cox regression analyses showed that ZBTB16 may be a prognostic factor for ES. The difference is statistically significant (*P* < 0.01) (Figure 5).

## 4. Discussion

Ewing's sarcoma (ES) is the second most common malignant bone tumor in children and adolescents [2]. At present, vincristine, doxorubicin, cyclophosphamide, ifosfamide, and etoposide are the standard chemotherapy regimens. However, the prognosis of patients is not optimistic [14, 15] and has little effect on patients with metastasis or recurrence [16]. Microarray technology provides a new direction for the treatment of patients with ES. Therefore, it is necessary to explore appropriate and effective measures to guide the prognosis of treatment.

The purpose of our microarray-based analysis is to identify novel biomarkers related to the prognosis of patients with ES. Based on GSE63157 and GSE17679 datasets, 1181 DEGs were screened and identified from the ES patient group and normal control group, including 364 upregulated genes and 817 downregulated genes. Then, the functional enrichment analysis was carried out, and the interaction between DEGs was discussed. GO and KEGG pathway enrichment analysis shows that DEGs are mainly involved in the muscle system process, muscle contract, structural constituent of muscle, electron transfer activity, diabetic cardiomyopathy, and cardiac muscle contraction. These biological functions are consistent with our knowledge of ES. Based on the MCODE plug-in computing the highest score module and survival analysis filtering, we chose the overlapping one gene as the hub gene according to the two modules.

Zinc-finger and BTB domain-containing 16 (ZBTB16), also known as promyelocytic leukemia zinc finger protein (PLZF), was first found in t(11;17) chromosome translocation, and fusion with retinoic acid receptor *α* (RARa) was identified [17]. ZBTB16 is an important mediator that regulates the expression of neuropeptides [18], ion channels [19], or various receptors [20], to influence the response to incoming signals and/or downstream neuron signals. ZBTB16 is expressed in various tissues and plays a variety of biological functions through the regulation of tissue-specific target genes, which is related to adipogenesis [21], myocardial remodeling [22], osteoblast differentiation [23], and obesity [24]. The enhanced expression of ZBTB16 can increase the number of mitochondria and the ability of respiration and uncoupling. Besides, ZBTB16 is associated with various tumors. ZBTB16 is a classic androgen receptor (AR) regulatory gene, which has antiproliferative activity in prostate cancer cells [25] and plays a role in inhibiting cancer by inhibiting the MAPK pathway [26]. Secondly, ZBTB16 may become a prognostic marker for breast cancer and hepatocellular carcinoma [27, 28].

Up to now, there is no literature report on the relationship between ZBTB16 and the prognosis of Ewing's sarcoma. Previous studies have found that ZBTB16 can be used to distinguish leiomyosarcomas from leiomyomas [29]. In addition, pathological studies have reported that ZBTB16 has high sensitivity and specificity in various tissue types of Primitive Neurological Tumor (PNET) [30]. This study verified the correlation between ZBTB16 and ES, and we need to further explore the relationship between ZBTB16 and the prognosis of ES. In our study, the expression of the ZBTB16 was downregulated in the ES group when compared to the control group. Survival analysis indicates that the high expression of the ZBTB16 was significantly higher than that in the low expression group, which further clarified that ZBTB16 was related to the prognosis of ES.

In a word, we confirmed that ZBTB16 provides valuable information for the prognosis of ES and may become a novel biomarker for the prognosis of ES patients. However, there are some limitations to this study. Due to the limited raw data, we did not consider the associations between ZBTB16 expression and clinicopathological factors (e.g., stage and grade) of patients with ES. Further research is needed to make up for the shortcomings of this article.

## 5. Conclusion

We identified ZBTB16 from the GEO database through bioinformatics analysis. Moreover, higher ZBTB16 expression patients can have a longer OS time. It may be the potential prognostic biomarker of ES. In addition, biological functions and mechanisms need to be studied further in ES.

## Figures and Tables

**Table 1:** GO and KEGG pathway enrichment analysis of DEGs in ES.

Term	Description	adj. *P* Val	Gene count
Biological processes			
BP	Muscle system process	2.28*E* − 37	110
BP	Muscle contraction	2.28*E* − 37	97
BP	Energy derivation by oxidation of organic compounds	9.88*E* − 36	84
BP	Actin-mediated cell contraction	3.57*E* − 27	49
BP	Muscle filament sliding	4.52*E* − 29	31
Cell component			
CC	Contractile fiber	2.45*E* − 57	97
CC	Myofibril	9.39*E* − 56	94
CC	Sarcomere	1.49*E* − 52	87
CC	I band	5.76*E* − 35	58
CC	Z disc	1.02*E* − 28	50
Molecular function			
MF	Actin binding	4.51*E* − 18	82
MF	Structural constituent of muscle	6.14*E* − 18	25
MF	Electron transfer activity	6.67*E* − 17	42
MF	Actin filament binding	8.16*E* − 11	43
MF	NADH dehydrogenase activity	3.12*E* − 09	18
KEGG pathway			
hsa05415	Diabetic cardiomyopathy	6.34*E* − 19	57
hsa04260	Cardiac muscle contraction	6.34*E* − 19	37
hsa05012	Parkinson disease	3.19*E* − 14	56
hsa04932	Nonalcoholic fatty liver disease	3.19*E* − 14	43
hsa04714	Thermogenesis	7.89*E* − 14	53
hsa00190	Oxidative phosphorylation	5.35*E* − 13	38
hsa05020	Prion disease	4.75*E* − 12	55
hsa05016	Huntington disease	4.67*E* − 11	57
hsa05208	Chemical carcinogenesis-reactive oxygen species	1.81*E* − 10	46
hsa05014	Amyotrophic lateral sclerosis	1.84*E* − 08	58
hsa05410	Hypertrophic cardiomyopathy	1.90*E* − 08	25
hsa05022	Pathways of neurodegeneration-multiple diseases	2.02*E* − 08	69
hsa05010	Alzheimer disease	2.18*E* − 08	58
hsa01200	Carbon metabolism	1.78*E* − 07	27
hsa05414	Dilated cardiomyopathy	3.06*E* − 07	24

DEGs = differentially expressed genes; GO = Gene Ontology; KEGG = Kyoto Encyclopedia of Genes and Genomes; BP = biological processes; CC = cell component; MF = molecular function.

## Data Availability

The data used to support the findings of this study are available from the corresponding author upon request.
